# Improved Left Ventricular Diastolic Function with Exercise Training in Hypertension: A Doppler Imaging Study

**DOI:** 10.1155/2011/497690

**Published:** 2011-01-23

**Authors:** Huan Zheng, Ming Luo, Yi Shen, Hong Fang

**Affiliations:** Tongji Hospital, Tongji University, 389 Xincun Road, Shanghai 200065, China

## Abstract

*Objective.* To study the effects of 6 months' exercise training on ventricular function in hypertensive patients. *Methods.* Both groups received routine anti-hypertensive pharmacological therapy and one received a 6 months' exercise program in addition. All patients underwent incremental cardiopulmonary exercise test and echocardiography in baseline and after 6 months. *Results.* (1) In 6 months' follow-up, Peak_VO2_, Power_max_ (max workload), AT (anaerobic threshold), VO_2AT_ (VO_2_ at anaerobic threshold), t_AT_ (time from beginning to anaerobic threshold) (*P* < .05), were increased in the exercise group. HR_rest_ (Heart rate at rest) was decreased (*P* < .05). LAVI (left atrial volume index), peak mitral filling velocities during early (E) and late (A) diastole E/A ratio, DT(deceleration time of the mitral E wave), IVST(Interventricular septum thickness in diastole), tissue Doppler indice Mean Ea/Aa ratio (*P* < .05) were also improved. (2) Correlation analysis: 4 variates had significant effect on change of Peak_VO2_ in the exercise group: age (*r* = −0.39), change of HR_rest_ (*r* = 0.59), change of E/A (*r* = 0.55), change of Mean Ea/Aa (*r* = 0.58); Through analyzing 2 groups patients' baseline values, their age (*β* = − 0.32), VO_2AT_ (*β* = 0.29), HR_rest_ (*β* = − 0.25), LAVI (*β* = − 0.24), E/A (*β* = 0.41) were found to be independent predictors of MeanEa/Aa. *P*-value under .05 was considered statistically significant. *Conclusion.* 6 months' exercise could enhance hypertensive patients' aerobic exercise level and diastolic function to a certain extent.

## 1. Introduction

It has been consistently shown that exercise training is a powerful nonpharmacological strategy to reduce blood pressure (BP) levels of hypertensive patients [1, 2] and to improve functional capacity, quality of life, and left ventricular ejection fraction (LVEF) of heart failure (HF) patients [3–5]. The progression of hypertensive cardiomyopathy towards HF includes serial left ventricular (LV) changes—LV remodeling, and LV hypertrophy. In presence of these LV geometric abnormalities, deep modification of LV diastolic properties occurs, which can precede alterations of LV systolic function. However, there is little research about the effects of exercise training on LV diastolic function in hypertensive patients. 

In this respect, the study was designed to assess LV geometric structure and diastolic function in hypertensive patients with or without exercise training. Tissue Doppler imaging (TDI), a highly sophisticated and superior technique compared with conventional echocardiography, was used for detailed analysis.

## 2. Methods

### 2.1. Study Population

The study was conducted on patients with hypertension who had attended the outpatient clinic of our institution. The participants consisted of 50 essential hypertensive patients whose hypertension duration were 10*∼*20 years and were under stable metabolic control for at least 1 year. They were randomly divided into 2 groups and stratified by age, sex, and body mass index (BMI). Both groups received routine pharmacological therapy and one received 6-month supervision exercise training in addition (exercise group). The 2 groups were similar in age, hypertension duration, LVEF, and pharmacological therapy. Patients with severe hypertension (supine systolic blood pressure [SBP] > 200 mmHg or diastolic blood pressure [DBP] > 110 mmHg), coronary artery disease (angina and/or ECG signs of ischemia on treadmill-exercise test), severe ventricular arrhythmia, atrioventricular block, severe reduction of LVEF (LVEF ≤ 45%), hypertrophic cardiomyopathy, valvular disease requiring surgery, pericarditis, diabetes mellitus, acute systemic illness or fever, severe renal dysfunction (i.e., creatinine >2.5 mg/dL), severe orthopedic problems that would prohibit exercise, and other metabolic problems such as acute thyroiditis, hypokalemia or hyperkalemia, or hypovolemia were excluded. 

The study was approved by the local Ethic Committee. All patients gave their written informed consent.

### 2.2. Cardiopulmonary Exercise Test

All patients underwent an incremental cardiopulmonary exercise test (CPET) on a bicycle ergometer. To stabilize respiratory exchange, patients were asked to remain still on the ergometer for at least 3 minutes before starting exercise. After a 1-minute warm-up period without any added workload, a ramp protocol of 15 W/min started and continued until exhaustion, including a 1-min cool-down period without added workload at the end. There were absolute and relative indications for terminating exercise test according to AHA Scientific Statement about Exercise Standard for Testing and Training [6]. We also adopted Borg's rate of perceived exertion [7] as our criteria; when the patient scored 16*∼*17, we stopped exercise. A 12-lead electrocardiogram was monitored continuously during the test, and cuff blood pressure was manually recorded every 2 minutes. Respiratory gas exchange measurements were obtained breath-by-breath with use of a computerized metabolic monitoring (Innocor 5.0, Innovision Cor., Denmark). Peak_VO2_ was recorded as the mean value of VO_2_ during the last 20 seconds of the test and expressed in ml × kg^−1^× min^−1^. Predicted Peak_VO2_ was determined by use of a sex, age, height, and weight-adjusted formula outlined by Wassermann et al. [8], and then the percentage of actual Peak_VO2_ in the predicted value (Peak_VO2_%) was calculated. Power_max_ was the maximal workload during exercise. The ventilatory anaerobic threshold (AT) was assessed mathematically; VO_2_ at AT (VO_2AT_) and time of duration from beginning to anaerobic threshold (t_AT_) were recorded.

### 2.3. Training Protocol

The patients in the exercise group attended the exercise training three times per week. Training sessions, performed under continuous electrocardiogram monitoring, were supervised by a cardiologist and a nurse. Each session was preceded by a 15-minute warming up and followed by a 15-minute cooling down. Exercise was performed for 30 minutes on a bicycle ergometer at VO_2AT_ (using target heart rate as a scale) achieved at the initial symptom-limited CPET, and the work load was adjusted according to the CPET results in 3-month followup.

### 2.4. Doppler Echocardiography

 All patients underwent complete transthoracic echocardiographic studies including two-dimensional, color flow, and spectral Doppler as well as TDI (Ultrasound Vivid 7, General Motor Cor., USA) at the beginning and after 6 months. Standard views, including the parasternal long axis, short axis at the papillary muscle level, and apical four, and two-chamber views were recorded. Left atrial volume index (LAVI) [9], left ventricular end-diastolic diameter (LVDd), left ventricular end-systolic diameter (LVSd), interventricular septum thickness in diastole (IVST), and posterior ventricular septum thickness in diastole (PVST) were collected. M-mode tracing of LV was obtained in the parasternal long-axis view with the cursor placed at the tip of the mitral valve leaflets. LVEF was estimated using a modified Simpson's biplane method. The mitral Doppler signals were recorded in the apical four-chamber view, with the Doppler sample volume placed at the tip of the mitral valve leaflets. The following parameters were obtained: early diastolic mitral inflow peak velocity (E), late diastolic mitral inflow peak velocity (A), their ratio (E/A), deceleration time of the mitral E wave (DT), isovolumic relaxation time (IVRT), and systolic velocity to diastolic velocity ratio by pulmonary veins (S/D). Tissue Doppler measurements were performed in the left lateral decubitus position during shallow respiration or end-expiratory apnea. Guided by the two-dimensional four-chamber view, a sample volume (length of the sample volume was 5 or 10 mm) was placed on the septum and lateral mitral anulus as well as medium segments of interventricular septum and lateral wall. The following respective parameters were obtained: peak diastolic velocities during early (Ea) and late (Aa) filling and MeanEa/Aa ratio calculated by averaging those four segmental Ea/Aa values. The echocardiographic studies were carried out by investigators blinded to whether the patients were in the exercise group or the control group.

### 2.5. Statistics

All data were normally distributed, and descriptive statistics were given in terms of mean value and standard deviation (SD). Comparisons between groups for continuous variables were made using independent *t*-tests where data were interval or ratio level. A *P* value under  .05 was considered statistically significant. Differences between the two groups and changes over time within each group were assessed by two-way repeated measures analysis of variance (ANOVA). Univariate correlation analysis of age, change of BMI, change of heart rate, and change of indices of diastolic filling with improvement of exercise capacity was performed in exercise group. Multiple regression analysis was carried out among those baseline values in 2 groups; mean Ea/Aa was regarded as an index of diastolic function and was entered in the statistical model as a dependent variable. All statistical analyses were performed using the software package SPSS, version 10.0 (SPSS Inc., Chicago, USA).

## 3. Results

All patients in both groups completed the 6-month program. No statistically significant differences were found at baseline between the exercise group and the control group in clinical, demographic, and echographic characteristics as well as pharmacological therapy (Tables 1 and 2).

In exercise group, we found increase in Peak_VO2_, Peak_VO2_%, Power_max_, AT, VO_2AT_, and t_AT_ and decrease in HR_rest_ at the end of 6 months, whereas in control group those indices remained unchanged (Table 3). Both groups' SBPrest, DBPrest, and BMI were unchanged (Table 3).

The results of echocardiographic examination were summarized in Table 4. There was no change in LVDd, LVSd, IVST, PVST, and LVEF in either group. Diastolic function measurements such as LAVI, E/A ratio, DT, IVRT, S/D, and mean Ea/Aa ratio were significantly different in the exercise group compared with the control group.

Univariate analysis showed that 4 variates had significant effect on improvement of exercise (change of Peak_VO2_ was regarded as dependent variable) in exercise group: age had an inverse correlation with change of Peak_VO2_, while change of HR, E/A, and Ea/Aa had a positive correlation with change of Peak_VO2_. age, VO_2AT_, HRrest, LAVI, and E/A and they remained in the regression equation (Tables 4 and 5).

## 4. Discussion

In our study we found that Peak_VO2_, Power_max_, VO_2AT_, AT, and t_AT_ all increased and HRrest decreased in patients who performed 6-month exercise training, while remaining unchanged in the control group. As to diastolic index, such as LAVI, E/A, DT, IVRT, S/D, and mean_Ea/Aa_, we observed that they were also improved in exercise group. What was important was the relationship among those clinical, exercise, and cardiographic parameters. Through regression analyzing we got that patients' age, HRrest, E/A, and Ea/Aa had significant effect on exercise capacity. Moreover, patients' age, VO_2AT_, t_AT_, LAVI, and E/A were predictors for mean Ea/Aa as well.

In our study we found that our patients' Peak_VO2_ was very low compared to normal values calculated by Wasserman's formula. Researchers in Copenhagen University reported that patients with a long history of hypertension and left ventricular hypertrophy could not reach expected exercise workload for their age, gender, and body, which was mainly because of reduction in vasodilatory performance and oxygen reserves. We thought that race difference might also be one of the reasons in our study.

As was already proved by Rinder et al., hypertensive patients with 6-month endurance exercise had a 15% increase in Peak_VO2_, which was considered to be the best measurement of cardiovascular fitness and exercise capacity, compared with those treated with thiazide, although exercise was less effective in reducing SBP than thiazide diuretics [9]. After 6-month training we observed that those exercise parameters improved, which suggested that aerobic metabolism was improved, because AT represented the point at which anaerobic metabolism started with production of lactic acid. Several previous researched studying heart failure patients have got similar conclusion years ago. Adamopoulos et al. [10] reported that after 8 weeks of bicycle training, phosphocreatine depletion was reduced, adenosine diphosphate concentrations were increased, and phosphocreatine recovery time was shorter. Those results suggested that with moderate exercise training the oxidative capacity of skeletal muscle could be improved. But neither SBP nor DBP was changed after 6-month exercise in our study, we thought that might be because their initial BP was in a relatively steady level as a result of a long history of drug control and 6-month exercise prescription probably would be a tiny contributor to the BP value.

As we know, the enhancement of diastolic left ventricular filling was a crucial determinant for the increase of cardiac output at exercise. Impairment of diastolic function could result in the inability to increase cardiac output adequately and can limit exercise capacity. Patients with isolated diastolic dysfunction due to arterial hypertension [11] demonstrate a maximal workload that was 2.5 metabolic equivalents (METs) lower than matched controls without diastolic dysfunction. Dumont et al. [12] found there was a negative correlation between exercise capacity and left ventricular stiffness in hypertensive patients. For the above-mentioned reasons, we investigated whether patients would get beneficial effects on diastolic function if their exercise capacities improved.

According to the use of echocardiography, LV diastolic function was graded as normal, abnormal relaxationpseudo, normal, and restrictive [13]. Doppler flow measurements could reveal a prolonged IVRT as a measure of a slow relaxation; E/A < 1 and DT > 220 ms; represented evidence of a slow early left ventricular filling. S/D < 1 indicated an elevated left atrial pressure resulting in a reincrease of the early mitral flow velocity and thus normalizing the E/A ratio. In addition, LAVI was valuable in detecting abnormal LV diastolic function. LAVI >26 mL/m^2^ was regarded as an independent predictor of diastolic dysfunction [14]. Furthermore, TDI indices of mitral annular diastolic velocities demonstrated a linear relation with regard to the progression of diastolic abnormalities [15]. Basal parts were generally more heavily affected, particularly the septal and inferior walls. The lateral wall and apical regions were more resistant to diastolic abnormalities [16]. So in our study we recorded Ea/Aa at four segments to get mean Ea/Aa to evaluate diastolic function.

After 6-month training we found exercise patients' HRrest declined and Peak_VO2_ and mean Ea/Aa increased. There was a close correlation among them, not only by univariate analysis but also by multivariate analysis. We thought lower HR might contribute to a longer diastolic filling time, improving ventricular function by a more operational Starling law.

It was previously reported that exercise training improved diastolic function in healthy subjects. Trained subjects demonstrated a higher increase of peak diastolic filling rate during exercise compared to untrained subjects [17]. An interventional study with 6 months of intensive aerobic endurance training in young and elderly subjects demonstrated improvement of diastolic function at rest and exercise in both groups [18]. In our study, exercise group showed significant improvement in diastolic index, although the ventricular geometric size was not changed yet. We had no idea whether we would get a favorable result about the change of LV size if our follow-up time was long enough. The causative mechanisms for the improvement in our patients after 6-month exercise were complicated. We hypothesized that one of the main reasons might be that moderate exercise training could reduce LV filling pressures. Giallauria et al. [19] found 3-month exercise induced a reduction in NT-pro-BNP and an increase in E/A in patients with LV dysfunction after myocardial infarction, and they thought those changes might be secondary to a reduction of LV systolic stress or afterload leading to an increase in atrioventricular pressure gradient in early diastole. Nevertheless, the decrease of LV systolic stress or afterload is likely due to several combined effects such as exercise effects on symptho/vagal balance [20].

## 5. Limitations

 We investigated the effect of 6-month exercise on LV diastolic function evaluating by echocardiography, without exploring the mechanism. Maybe we could look for some other evidence to support our conclusion more effectively, such as serum BNP level, because the measurement of BNP has emerged recently as a highly sensitive and accurate method for the detection of LV diastolic dysfunction [21].

## 6. Conclusion

In conclusion, we have demonstrated that the aerobic exercise level and the diastolic function are improved in patients with exercise training compared with the control participants, although the mechanism is unclear, which needs further studies.

## Figures and Tables

**Table 1:** Baseline clinical, morphological, and echographic characteristics of trained patients and untrained controls.

	Exercise group	Control group	*P* value
	*n* = 25	*n* = 25	
Age (years)	64.6 (3.7)	65.0 (3.1)	.641
Male/female	16/9	15/10	.329
BMI (kg/m^2^)	24.7 (3.6)	23.8 (3.0)	.436
Hypertension duration (years)	13.2 (3.5)	12.9 (3.8)	.712
HRrest (beats/min)	74.5 (6.1)	72.6 (7.4)	.193
SBPrest (mmHg)	138 (14)	140 (13)	.384
DBPrest (mmHg)	78 (13)	76 (15)	.211
LAVI (ml/m^2^)	35.4 (2.1)	36.0 (1.8)	.431
LVDd (mm)	47.4 (5.5)	48.1 (3.3)	.828
LVSd (mm*）*	32.4 (2.6)	32.8 (2.4)	.783
IVST (mm)	10.8 (0.9)	11.1 (0.7)	.672
PVST (mm)	10.3 (0.4)	10.4 (0.3)	.532
LVEF (%)	54.5 (5.5)	55.0 (4.9)	.471

BMI: body mass index; HRrest: heart rate at rest; SBPrest: systolic blood pressure at rest; DBPrest: diastolic blood pressure at rest; LAVI: left atrial volume index; LVDd: left ventricular end-diastolic diameter; LVSd: left ventricular end-systolic diameter; IVST: interventricular septum thickness in diastole; PVST: posterior ventricular septum thickness in diastole; LVEF: left ventricular ejection fraction.

**Table 2:** Pharmacological therapy at baseline and after 6 months in the 2 patient groups.

	Exercise group		Control group		Differences in changes between groups
	*n* = 25		*n* = 25		
	Baseline	6 months	Baseline	6 months	*P* value
Fosinopril	12.2 (3.8)	11.8 (3.9)	11.9 (4.1)	12.5 (5.0)	.732
(mg/d) [*n*]	[17]	[17]	[16]	[16]	
Benazepril	8.3 (2.3)	7.8 (2.2)	8.4 (2.4)	9.0 (2.3)	.819
(mg/d) [*n*]	[6]	[6]	[7]	[7]	
Bisoprolol	3.8 (1.5)	3.4 (1.6)	4.0 (1.3)	4.3 (1.5)	.328
(mg/d) [*n*]	[14]	[14]	[5]	[5]	
Nitrendipine	13.6 (4.3)	14.1 (4.1)	14.0 (3.9)	13.5 (4.2)	.136
(mg/d) [*n*]	[10]	[10]	[10]	[10]	
Felodipine	6.9 (2.4)	6.9 (2.4)	7.1 (2.2)	7.1 (2.2)	1
(mg/d) [*n*]	[9]	[9]	[9]	[9]	
Hydrochlorothiazide	42.6 (9.5)	41.9 (8.8)	42.5 (9.4)	42.5 (9.4)	.739
(mg/d) [*n*]	[7]	[7]	[7]	[7]	
Aspirin	100	100	100	100	1
(mg/d) [*n*]	[25]	[25]	[25]	[25]	

Values in parenthesis refer to total daily dosage; (*n*) refers to the number of patients consuming the drug.

**Table 3:** Cardiopulmonary exercise test parameters and clinical characteristics at baseline and after 6 months in the 2 patient groups.

	Exercise group		Control group		*P-*value
	*n* = 25		*n* = 25		
	Baseline	6 months	Baseline	6 months	
Peak_VO2_ (ml × kg^−1^× min^−1^)	14.1 (1.5)	16.5 (2.1)	13.9 (1.6)	13.6 (2.2)	.008
Peak_VO2_%	50.3 (2.1)	60.4 (2.6)	47.6 (3.8)	46.8 (4.2)	.013
Power_max_ (W)	67.4 (8.2)	76.9 (6.3)	66.8 (7.8)	67.3 (8.2)	.029
AT (W)	61.3 (6.9)	71.0 (7.7)	60.8 (7.4)	60.6 (8.1)	.017
VO_2AT_ (ml × kg^−1^× min^−1^)	11.2 (2.9)	13.2 (2.7)	11.4 (3.1)	10.9 (2.8)	.003
t_AT_ (s)	6.7 (0.8)	8.2 (1.0)	7.0 (0.7)	6.9 (0.9)	.011
SBP_rest_ (mmHg)	138 (14)	136 (15)	140 (13)	138 (14)	.271
DBP_rest_ (mmHg)	78 (13)	80 (15)	76 (15)	75 (16)	.295
HR_rest_	74.5 (6.1)	71.6 (5.5)	72.6 (7.1)	72.9 (6.4)	.024
BMI (Kg/m^2^)	24.7 (3.6)	23.8 (4.0)	23.8 (3.0)	23.7 (3.1)	.442

Peak_VO2_: peak oxygen cousumption; Peak_VO2_%: the percentage of actual Peak_VO2_ in the predicted value; Power_max _: maximal workload; AT: ventilatory anaerobic threshold; VO_2AT_: oxygen consumption at anaerobic threshold; t_AT_: time from beginning to anaerobic threshold; SBPrest: systolic blood pressure at rest; DBPrest: diastolic blood pressure at rest; HRrest: heart rate at rest; BMI: body mass index.

**Table 4:** Doppler echocardiographic parameters at baseline and after 6 months in the 2 patient groups.

	Exercise group		Control group		*P-*value
	*n* = 25		*n* = 25		
	Baseline	6 months	Baseline	6 months	
LAVI (ml/m^2^)	35.1 (4.2)	32.3 (3.8)	35.7 (3.9)	35.9 (4.2)	.017
LVDd (mm)	47.4 (5.5)	46.9 (6.1)	48.1 (3.3)	48.3 (4.0)	.592
LVSd (mm)	32.4 (2.6)	33.1 (2.8)	32.8 (2.4)	33.1 (3.1)	.273
IVST (mm)	10.8 (0.9)	10.5 (1.0)	11.1 (0.7)	10.9 (0.9)	.269
PVST (mm)	10.3 (0.4)	10.2 (0.5)	10.4 (0.3)	10.4 (0.6)	.727
LVEF (%)	54.5 (5.5)	55.1 (6.2)	55.0 (4.9)	54.8 (4.6)	.614
E/A	0.75 (0.07)	0.88 (0.06)	0.72 (0.05)	0.70 (0.07)	.004
DT (ms)	220 (14)	242 (18)	226 (20)	218 (19)	.018
IVRT (ms)	109 (12)	89 (11)	106 (14)	109 (13)	.037
S/D	1.4 (0.2)	1.1 (0.3)	1.5 (0.2)	1.2 (0.2)	.008
Ea/Aa [1]	0.87 (0.26)	1.12 (0.28)	0.90 (0.24)	0.96 (0.30)	.019
Ea/Aa [2]	0.92 (0.18)	1.20 (0.24)	0.89 (0.17)	0.87 (0.25)	.006
Ea/Aa [3]	0.85 (0.17)	1.05 (0.23)	0.86 (0.23)	0.86 (0.19)	.028
Ea/Aa [4]	0.96 (0.16)	1.21 (0.18)	0.96 (0.27)	0.93 (0.29)	.036
Mean Ea/Aa	0.89 (0.23)	1.19 (0.21)	0.91 (0.23)	0.90 (0.24)	.011

LAVI: left atrial volume index; LVDd: left ventricular end-diastolic diameter; LVSd: left ventricular end-systolic diameter; IVST: interventricular septum thickness in diastole; PVST: posterior ventricular septum thickness in diastole; LVEF: left ventricular ejection fraction; E: early diastolic mitral inflow peak velocity; A: late diastolic mitral inflow peak velocity; DT: deceleration time of the mitral E wave; IVRT: isovolumic relaxation time; S/D: systolic velocity to diastolic velocity ratio by pulmonary veins; Ea: early diastolic myocardial peak velocity; Aa: late diastolic myocardial peak velocity; Ea/Aa [1], Ea/Aa [2], Ea/Aa [3], and Ea/Aa [4] refer to Ea/Aa ratio at septum mitral annulus, lateral mitral annulus, interventricular medium septum, and lateral medium wall, respectively; meanEa/Aa = (Ea/Aa [1] + Ea/Aa [2] + Ea/Aa [3] + Ea/Aa [4])/4.

**Table 5:** Univariate correlation in exercise group.

	Δ Peak_VO2_
Age	−0.39*
Hypertension-duration	−0.46
Δ BMI	0.14
Δ SBPrest	0.22
Δ DBPrest	0.31
Δ HR	0.59*
Δ LAVI	0.20
Δ LVDd	0.28
Δ LVSd	0.49
Δ IVST	0.17
Δ PVST	0.27
Δ LVEF	0.34
Δ E/A	0.55*
Δ DT	0.41
Δ IVRT	0.34
Δ MeanEa/Aa	0.58*

*P < .05, Δ means change of those values after 6 months.

**Table 6:** Multi-variate regression analysis for Mean Ea/Aa.

	*β*
Age	−0.32*
VO_2AT_	0.29*
HRrest	−0.25*
LAVI	−0.24*
E/A	0.41*

*P < .05.
